# *Desulfovibrio* Bacteria Are Associated With Parkinson’s Disease

**DOI:** 10.3389/fcimb.2021.652617

**Published:** 2021-05-03

**Authors:** Kari E. Murros, Vy A. Huynh, Timo M. Takala, Per E. J. Saris

**Affiliations:** ^1^ Neurological Outpatient Clinic of Terveystalo Healthcare, Helsinki, Finland; ^2^ Department of Microbiology, Faculty of Agriculture and Forestry, University of Helsinki, Helsinki, Finland

**Keywords:** hydrogen sulfide, magnetite, alpha-synuclein (α-Syn), gut *Desulfovibrio* bacteria, Parkinson’s disease (PD)

## Abstract

Parkinson’s disease (PD) is the most prevalent movement disorder known and predominantly affects the elderly. It is a progressive neurodegenerative disease wherein α-synuclein, a neuronal protein, aggregates to form toxic structures in nerve cells. The cause of Parkinson’s disease (PD) remains unknown. Intestinal dysfunction and changes in the gut microbiota, common symptoms of PD, are evidently linked to the pathogenesis of PD. Although a multitude of studies have investigated microbial etiologies of PD, the microbial role in disease progression remains unclear. Here, we show that Gram-negative sulfate-reducing bacteria of the genus *Desulfovibrio* may play a potential role in the development of PD. Conventional and quantitative real-time PCR analysis of feces from twenty PD patients and twenty healthy controls revealed that all PD patients harbored *Desulfovibrio* bacteria in their gut microbiota and these bacteria were present at higher levels in PD patients than in healthy controls. Additionally, the concentration of *Desulfovibrio* species correlated with the severity of PD. *Desulfovibrio* bacteria produce hydrogen sulfide and lipopolysaccharide, and several strains synthesize magnetite, all of which likely induce the oligomerization and aggregation of α-synuclein protein. The substances originating from *Desulfovibrio* bacteria likely take part in pathogenesis of PD. These findings may open new avenues for the treatment of PD and the identification of people at risk for developing PD.

## Introduction

In Parkinson’s disease (PD), intestinal symptoms such as constipation often precede the appearance of motor symptoms, suggesting that an etiological agent may be present in the intestine. Based on neuropathological findings, Braak and colleagues proposed in 2003 that PD is caused by an intestinal pathogen capable of passing through the gut mucosal barrier and traveling through enteric neurons before finally entering the central nervous system *via* the vagus nerve (Braak et al., 2003). In support of this view, Lewy bodies and Lewy neurites containing phosphorylated α-synuclein protein (α-Syn), the classic neuropathological hallmarks of PD, can be found in both the central nervous system and the enteric nervous system (Barrenschee et al., 2017). Additionally, injected forms of α-Syn are transported to the lower brainstem *via* the vagus nerve in rats (Holmqvist et al., 2014). It is proposed that the production of toxins and metabolites by gut microbes play a critical role in the pathogenesis of PD and that gut enteroendocrine cells serve as sites for the initial emergence of pathologic α-Syn (Chandra et al., 2017).

Since 2015, changes in gut microbiota compositions in PD have been found in several large-scale case-control studies (Scheperjans et al., 2015; Shen et al., 2021). Composition changes concerning the phylum level are difficult to establish due to high variability in the relative abundances of bacterial phyla in PD studies (Chiang and Lin, 2019). Concerning the family level of bacteria, a recent meta-analysis on fourteen case-control studies showed, by 95% confidence interval analysis, significantly increased relative abundances of *Verrucomicrobiaceae, Bifidobacteriaceae*, and *Christesenellaceae* in PD gut microbiota (Shen et al., 2021). At the genus level, opportunistic pathogens including *Porphyromonas*, *Prevotella* and *Corynebacterium* were found to be elevated in PD in a microbiome-wide association study (Wallen et al., 2020). A limitation of these studies is that assessing relative changes in microbiota composition, rather than absolute quantitative changes, limits the chances of identifying disease-associated ecosystem configurations (Vandeputte et al., 2017; Boertien et al., 2019; Haikal et al., 2019). In this respect, the dynamics of *Desulfovibrio* bacteria (*DSV*) are not fully captured by studies focusing on the relative bacterial changes in PD gut microbiota. One case-control study offered a broad indication of *DSV* dynamics, finding that the relative abundance of bacteria in the *Desulfovibrionaceae* family was elevated in PD patient microbiota (Lin et al., 2018). *DSV* have several interesting characteristics that lend themselves toward a potential role in PD pathogenesis and warrant further investigation. *DSV* are sulfate-reducing bacteria (SRB), commonly found in the environment and the human intestine with the potential to cause infections in humans (Loubinoux et al., 2002; Goldstein et al., 2003). As such, *DSV* produce hydrogen sulfide (H_2_S), a metabolite known to influence cell signaling in neuronal cells at low concentrations and pose serious toxicity at higher concentrations (Carbonero et al., 2012; Panthi et al., 2018; Haouzi et al., 2020). H_2_S has been observed to release mitochondrial cytochrome c into the cytosol, where the cytochrome is able to form α-Syn radicals and thereby initiate α-Syn oligomerization (Guo et al., 2015; Kumar et al., 2016). Further, H_2_S can interfere with iron metabolism by increasing iron levels in the cytosol (Cassanelli and Moulis, 2001; Hälldin and Land, 2008), an event potentially inducing α-Syn aggregate formations (Joppe et al., 2019). *DSV* have been found to colonize the mucus gel layer of the colon (Nava et al., 2012; Earley et al., 2015). Therefore, the α-Syn-expressing enteroendocrine cells located in the gut wall, in close proximity to intestinal *DSV*, may be especially vulnerable to the toxic effects of H_2_S and serve as a seeding point for α-Syn aggregation in the nervous system. Additionally, *DSV* have the ability to reduce ferric iron to ferrous iron by employing a periplasmic [FeFe]-hydrogenase enzyme, present in practically all *DSV*, conferring the ability to produce magnetite (Fe_3_O_4_) (Chistyakova et al., 2004; Park et al., 2008; Pereira et al., 2011). Uncoated magnetite nanoparticles have been reported to accelerate α-Syn aggregation and are considered to be involved in PD pathogenesis (Joshi et al., 2015; Murros et al., 2019). As *DSV* are found in the gut microbiota of humans and are capable of producing extracellular magnetite and H_2_S, both of which induce oligomerization and aggregation of α-Syn, it is rational to suspect that there may be a correlation between these bacteria and PD. Here, we employed a targeted PCR-based approach to determine absolute quantitative changes in the levels of *DSV* bacteria between PD patients and healthy controls.

## Materials and Methods

### Research Subjects, Sample Collection, and Ethical Issues

The study participants consisted of 20 PD patients and 20 healthy controls. PD patient were recruited from the patient material of the Neurology Outpatient Clinic of Terveystalo Healthcare, Kamppi, Helsinki. The control group consisted of 10 spouses and 10 non-spouses of PD patients. As a prerequisite, selected PD patients had to fulfill the clinical features of the UK Parkinson’s Disease Society Brain Bank diagnostic criteria (Hughes et al., 1992). Furthermore, PD patient disease progression was scored using the Hoehn and Yahr scale as an estimate of the clinical stage of PD (Hoehn and Yahr, 1967). Symptoms or signs of parkinsonism were exclusion criteria for the controls. Exclusion criteria for both groups included cognitive disturbance (Mini-Mental State Examination points < 25) and a history of antibiotic use within 3 months prior to the date of fecal sampling. Fecal samples were collected by donors in sealed polypropylene containers and subsequently frozen and stored at -75°C until further analyses. The study was approved by the Ethics Committee of Helsinki and the Uusimaa Health District area of Finland, and all procedures were in accordance with the relevant regulations. Each study participant also provided written informed consent.

### Bacterial Strains and Culture Conditions

Two collection strains, *D. desulfuricans* MB (DSM 6949) and *D. vulgaris* DSM 644, were obtained from the Leibniz Institute DSMZ-German Collection of Microorganisms and Cell Cultures, Braunschweig, Germany. Liquid Postgate medium (DSMZ medium 63) for bacterial culture was made anoxic by sparging with nitrogen gas for 1 hour at 80°C prior to autoclavation. The medium was aliquoted into Hungate-type tubes and bacterial inoculation was performed in an anaerobic workstation (Don Whitley Scientific, West Yorkshire, UK). The bacteria were cultured anaerobically at 37°C. Anaerobic conditions inside the anaerobic jar were maintained using the Microbiology Anaerocult^®^ A system (Merck KGaA, Darmstadt, Germany) and indicated by an Anaerotest^®^ strip (Merck KgaA). Ferrous sulfide (black precipitate) formation after two to seven days was used as an indicator for bacterial growth.

### DNA Techniques

DNA from the fecal samples was extracted using the Stool DNA Isolation Kit (Norgen Biotek, Ontario, Canada). The bacterial DNA of *D. desulfuricans* MB (DSM 6949) and *D. vulgaris* DSM 644, for use as positive controls, were isolated using the MagAttract HMW DNA Kit (Qiagen GmbH, Hilden, Germany). PCR products were purified using the SanPrep Column PCR Product Purification kit (BBI Life Sciences, Shanghai, China). Gel electrophoresis was performed in 0.9% or 1.5% (w/v) agarose gel containing 0.1 μg/ml ethidium bromide, followed by visualization under UV light. The size markers used were 100 bp GeneRuler DNA ladder or 1 kb GeneRuler DNA ladder (Thermo Fisher Scientific, Waltham, MA, USA).

### Primers and PCR Conditions

A pair of universal primers targeting the bacterial 16S rRNA gene were used to validate the success of DNA isolation from fecal samples. Primers for detecting periplasmic [FeFe]- hydrogenase gene were designed by multiple sequence alignment of the periplasmic [FeFe]- hydrogenase large subunit (*hydA*) genes of different *Desulfovibrio* spp. The primer specificity was analyzed using the Primer-BLAST webtool by NCBI (https://www.ncbi.nlm.nih.gov/tools/primer-blast/). The 16S rRNA gene primer sequences used for specific detection of the *Desulfovibrio* genus and species including *Desulfovibrio desulfuricans*, *D. fairfieldensis*, *D. piger*, and *D. vulgaris* were obtained from previous studies. All primers used in this study are listed in **Table 1**.

**Table 1:** Primers used in the study.

Primer name	Sequence 5’ → 3’	Target	Amplified region (nt)	PCR product size (bp)	Source
**pA**	AGAGTTTGATCCTGGCTCAG	Bacterial 16S rRNA	8-929	922	Edwards et al., 1989
**pE’**	CCGTCAATTCCTTTGAGTTT				
**DSV691-F**	CCGTAGATATCTGGAGGAACATCAG	*Desulfovibrio* genus	704-839	136	Fite et al., 2004
**DSV826-R**	ACATCTAGCATCCATCGTTTACAGC	16S rRNA			
**27K-F**	CTGCCTTTGATACTGCTTAG	*D. desulfuricans* MB	630-1025	396	Loubinoux et al., 2002
**27K-R**	GGGCACCCTCTCGTTTCGGAGA	(DSM 6949) 16S rRNA			
**Fair-F**	TGAATGAACTTTTAGGGGAAAGAC	*D. fairfieldensis*	181-714	534	Loubinoux et al., 2002
**P687-R**	GATATCTACGGATTTCACTCCTACACC	(ATCC 700045) 16S rRNA			
**Pig-F**	CTAGGGTGTTCTAATCATCATCCTAC	*D. piger*	460-714	255	Loubinoux et al., 2002
**P687-R**	GATATCTACGGATTTCACTCCTACACC	(ATCC 29098^T^) 16S rRNA			
**Dv1F**	AAGACCTTCCCGAAAAGGAA	*D. vulgaris*	1004-1158	155	Chakraborty et al., 2011
**Dv1R**	ACCAGAGTGCCCAGCATTAC	(DSM 644) 16S rRNA			
**Pair *hydA*1**					
***hydA*-F(a) *hydA*-R(a)**	GACGTGACCATCTGGGAAGA	Periplasmic [FeFe]-	448-1127	680	This study
**Pair *hydA* 2**	CAGGCCATGAATTCGATGAA	hydrogenase gene of *DSV*			
***hydA*-F(b)**	ACCGTCTCCATCATGCCCTG	Periplasmic [FeFe]-	676-1127	462	This study
***hydA*-R(a)**	CAGGCCATGAATTCGATGAA	hydrogenase gene of *DSV*			
**Pair *hydA* 3**					
***hydA*-F(a)**	GACGTGACCATCTGGGAAGA ¨	Periplasmic [FeFe]-	448-695	248	This study
***hydA*-R(b)**	CAGGGCATGATGGAGACGGT	hydrogenase gene of *DSV*			
**AmpF**	GCACTTTTCGGGGAAATG	*amp^R^*-*ori* fragment		2020	This study
**OriR**	CAGTCGGGAAACCTGTCGTG				

A standard PCR procedure was followed in both conventional and quantitative PCR according to the manufacturer’s protocol for Phusion High-Fidelity DNA polymerase (Thermo Fisher Scientific). Specifically, each 20 µl reaction comprised of 1× Phusion HF buffer (Thermo Fisher Scientific), 0.2 mM dNTP mix (Thermo Fisher Scientific), 0.5 µM of each primer, 1 U of Phusion High-Fidelity DNA polymerase (Thermo Fisher Scientific), and DNA. The thermal cycle was set as follows: 98°C for 30 secs followed by 30-40 cycles of denaturing at 98°C for 10 secs, annealing at 55-62°C, depending on the primers used, for 10 secs and elongation at 72°C for 20 secs, continuing with 72°C for 5 min and final 4°C for 15 min. Reaction mixtures contained approximately 170 ng of total DNA extracted from fecal samples or 20 ng of bacterial genomic DNA or water as negative control.

The PCR products were checked by gel electrophoresis, purified, and sequenced (Institute of Biotechnology, University of Helsinki, Finland), followed by comparison to the NCBI GenBank database for analysis.

### Cloning of 16S rRNA and *hydA* Gene PCR Products

Vector pHelix1 (Roche Diagnostics GmbH, Mannheim, Germany) was amplified with the primers AmpF and OriR to obtain an amplicon containing only the ampicillin resistance gene and the origin of replication (*amp^R^*-*ori*). The amplicons were purified and checked by gel electrophoresis.

The purified PCR products of the 16S rRNA gene of the four *DSV* species and the *hydA* amplicons of *D. desulfuricans* MB, after being verified by sequencing, were phosphorylated with T4 Polynucleotide Kinase (Thermo Scientific) and ligated with the *amp^R^-ori* fragment using T4 DNA ligase (Thermo Scientific). The ligation mixtures were introduced into competent *E. coli* XL1-Blue cells (Agilent Technologies, Santa Clara, CA, USA) by electroporation with pulse 2.5 kV, 200 Ω and 25 μFD (Zabarovsky and Winberg, 1990). The transformed cells were plated on LB agar plates containing 100 μg/ml ampicillin. Plasmids were isolated using a SanPrep Column Plasmid Mini-preps kit (BBI Life Sciences), and the correct constructs were confirmed by PCR with corresponding insert primers and gel electrophoresis. Plasmids with correct inserts were used as standards in quantitative real-time PCR.

### Quantitative Real-Time PCR (qPCR)

Samples which were deemed to contain both *DSV* and *hydA* were selected for bacterial quantification using a QuantStudio 3 Real-Time PCR System (Thermo Fisher Scientific). SYBR™ Green I (FMC BioProducts, Rockland, ME, USA) at a final concentration of 0.1× was used as a fluorescent dye. Constructed plasmids carrying 16S rRNA or *hydA* fragments were used as standards. Reaction mixtures contained approximately 70 ng of total DNA extracted from fecal samples or standards with final amount ranging from 2×10^3^ to 2×10^7^ copies. Reaction mixtures containing 70 ng of extracted DNA from *DSV-*bacteria-negative healthy controls stool were also created and used to construct standard curves. Reactions containing sterile Milli-Q water instead of DNA were used as negative controls. Each PCR reaction was performed in technical triplicates. The specificity of the amplification was assessed by melt curve analysis. Gel electrophoresis was performed to confirm these predictions.

### Statistical Analysis

IBM SPSS Statistics 20 software was used to analyze the results. A Fisher’s exact test was used to examine the association between the presence of *DSV* bacteria or the *DSV*-specific *hydA* gene and Parkinson’s disease. The strength of the association was analyzed by Phi and Cramer’s V test with an outcome value greater than 0.25 indicating a strong relationship. A Mann-Whitney U test was used to compare *DSV* quantities between PD patients and healthy controls, as well as between patients with high and low levels of disease progression. The significance threshold was set at 0.05. All tests were two-sided.

## Results

### Basic Characteristics of Study Participants

The PD group was well-matched with the control group with regard to age, gender, and body mass index. Fifteen PD patients presented with idiopathic hyposmia, and fourteen patients suffered from chronic constipation (fewer than three bowel movements a week) in contrast to none in the control group (**Table 2**).

**Table 2:** Clinical characteristics of patients and controls.

	Patients (n = 20)	Controls (n = 20)
Age in years, median (range)	70.0 (58-80)	68.5 (54-79)
Gender, male (%)	60	40
Body Mass Index, kg/m^2^, median (range)	25.0 (18.3-33.0)	23.5 (18.4-29.4)
Years from PD diagnosis, median (range)	10 (2-26)	–
Hoehn & Yahr stage > 2, prevalence (%)	55	–
Daily levodopa dose (mg), median (range)	525 (200-1100)	–
Dopamine agonist users (%)	70	–
Probiotic users (%)	10	5
Hypertensive, on medication (%)	25	20
Idiopathic hyposmia, prevalence (%)	75	0
Constipation, prevalence (%)	70	0
Current smokers (%)	0	0

### Association of *Desulfovibrio* spp., *hydA*, and Parkinson’s Disease

In order to establish a relationship between the presence of *Desulfovibrio* spp. in the human intestinal tract and the development of PD, we performed conventional PCR to specifically detect *DSV* within fecal samples obtained from PD patients and healthy controls. All PCR products from control laboratory strains and fecal samples were tested for target specificity by gel electrophoresis and subsequent sequencing. By sequencing, it was confirmed that the species-level primers specific to *D. desulfuricans*, *D. fairfieldensis*, *D. piger*, and *D. vulgaris* showed high specificity, as they amplified 16S rRNA fragments of expected sizes only from the corresponding bacterial species. The genus-level primers to detect *Desulfovibrio* also amplified fragments of correct sizes from the fecal samples, but some amplicons were later determined to not be *Desulfovibrio* DNA by sequencing. Thus, as the genus-level primers were not specific, they were excluded from subsequent experiments.

In total, sixteen PD patients (80%) and eight healthy controls (40%) were positive with species specific *DSV* PCR (**Table 3**). Some samples from healthy controls were detected with more than one species of *DSV*. In PD patients, *D. desulfuricans*, *D. fairfieldensis*, and *D. piger* were found, whereas for the healthy controls group, all four examined species were detected (**Table 3**). Statistical analysis revealed a strong association between the presence of *DSV* and PD (*P* = 0.022, Fisher’s exact test, Phi value = 0.408). Due to the non-specificity of the genus-level 16S rRNA primers, three additional primer pairs targeting the *DSV*-specific [FeFe]- hydrogenase (*hydA*) gene were designed and tested as a proxy for detecting a wider range of *DSV* species, as well as an indicator of putative magnetite production in participants’ microbiota. Sequencing of the obtained test-PCR products showed that only *hydA*-primer pair 1 specifically amplified *DSV*-*hydA* fragments of correct sizes, and thus those primers were selected for further experiments. As a result, *hydA* was detected in fecal samples from all 20 PD patients (100%) and 13 healthy controls (65%).The PCR results were confirmed by sequencing. In comparison to the results from strain/species-specific primers, an additional four patients and five healthy controls were determined to possess *DSV* bacteria with the *hydA*-specific primers. The presence of the *DSV*-specific [FeFe]- hydrogenase gene was strongly correlated with PD (*P* = 0.008, Fisher’s exact test, Phi value = 0.461).

**Table 3:** Summary of the PCR detection and quantification of *Desulfovibrio* spp. from patients and healthy individuals.

Parkinson’s group (n=20)				Control group (n=20)			
Gender	*DSV* species detected by PCR	*DSV* quantity (bacteria/g feces)	*hydA*	Gender	*DSV* species detected by PCR	*DSV* quantity (bacteria/g feces)	*hydA*
M*	*D. piger*	3.3×10^7^	+	M	*D. fairfieldensis*	1.9×10^6^	+
M*	*D. fairfieldensis*	2.6×10^7^	+	M	*D. fairfieldensis*	1.0×10^6^	+
M*	*D. fairfieldensis*	2.6×10^7^	+	M	*D. fairfieldensis*	5.6×10^5^	+
M*	*D. fairfieldensis*	1.6×10^7^	+	F	*D. desulfuricans*	1.9×10^5^	+
M*	*D. desulfuricans*	7.3×10^6^	+	M	*D. desulfuricans*, *D. vulgaris*	7.8×10^4^	+
F*	*D. fairfieldensis*	4.2×10^6^	+	F	*D. desulfuricans*	5.1×10^4^	+
M*	*D. piger*	2.2×10^6^	+	M	*D. desulfuricans*, *D. fairfieldensis*, *D. piger*, *D. vulgaris*	2.6×10^4^	+
F	*D. desulfuricans*	1.2×10^4^	+	M	*D. piger*	1.3×10^4^	+
M	*D. desulfuricans*	4.4×10^3^	+	M	None		+
F	*D. desulfuricans*	4.1×10^3^	+	F	None		+
F*	*D. desulfuricans*	2.1×10^3^	+	F	None		+
F*	*D. desulfuricans*	< 2.0×10^3^	+	M	None		+
F*	*D. desulfuricans*	< 2.0×10^3^	+	F	None		+
M*	*D. desulfuricans*	< 2.0×10^3^	+	M	None		None
F	*D. desulfuricans*	< 2.0×10^3^	+	F	None		None
M	*D. desulfuricans*	< 2.0×10^3^	+	F	None		None
M	None		+	M	None		None
M	None		+	F	None		None
M	None		+	F	None		None
F	None		+	M	None		None

The cases are displayed by decreasing *DSV* quantities. M, male; F, female. Asterisks indicate patients with level of disability exceeded 2.0 points based on Hoehn-Yahr classification.

### Quantity of *DSV* Bacteria in Human Feces and Parkinson’s Disease

To assess whether there is a difference in *DSV* quantity between healthy controls and PD patients, quantitative real-time PCR was carried out to determine the bacterial amount in the *DSV*- and *hydA*-positive fecal samples from both groups. Standard curves were constructed for every PCR reaction using serial dilutions of the constructed standard plasmids with known copy numbers. The qPCR products were checked by gel electrophoresis. The results revealed that PD patients had significantly higher amounts of *DSV* in their feces than healthy controls (*P* = 0.044, Mann-Whitney U-test). Although most PD patients had relatively low levels of *DSV* (< 10^5^ bacteria/g feces), the quantity reached to as high as 3.3×10^7^ bacteria/g feces. In the healthy controls group, the maximum *DSV* level was approximately 1.9×10^6^ bacteria/g feces (**Table 3**).

In addition, we examined whether the amount of *DSV* present in PD patient fecal samples correlated with the severity of the disease. The level of disability exceeded 2.0 points in 11 patients based on the Hoehn-Yahr classification system. Notably, all seven patients with *DSV* loads higher than any of the healthy controls belonged to this category. Furthermore, the eleven patients with a more severe disability of PD had a significantly higher amount of *DSV* bacteria than the nine patients that were classified below 2.0 points under the Hoehn-Yahr system (*P* = 0.009, Mann-Whitney U-test). We also investigated the *DSV* levels between subjects experiencing constipation (n=14) and not experiencing constipation (n=26). The statistical result suggested that the *DSV* amount was significantly higher in the group suffering from constipation (*P* = 0.036, Mann-Whitney U-test). As idiopathic hyposmia was very prevalent among PD patients, Mann-Whitney U test was performed to compare *DSV* quantity between individuals who suffered from hyposmia (n=15) and those who did not (n=25). Statistically, *DSV* bacteria were significantly more abundant in patients with hyposmia (*P* = 0.009).

## Discussion

Our results established a significant correlation between *DSV* bacteria and PD. The quantity of *DSV* bacteria in fecal samples correlated with the severity of the disease, and higher amounts of *DSV* were found in PD samples compared to control samples. All fecal samples of PD patients were positive for the *DSV*-specific [FeFe]-hydrogenase gene. *DSV* bacteria, *D. desulfuricans, D. fairfieldensis*, and *D. piger*, were significantly more common in PD samples than in control samples. Previous attempts to correlate *DSV* abundance with different intestinal diseases have failed to show correlations (Zinkevich and Beech, 2000; Fite et al., 2004; Scanlan et al., 2009). In our study, all PD patients harbored *DSV*, but as the primers used for *hydA* detection were not suitable for qPCR (results not shown), we cannot exclude the possibility that the patients with low levels of the four examined *DSV* species may have high levels of other *DSV* species. As 20% of the PD patients had unknown *DSV* species, these bacteria must be isolated and characterized to enable the development of primers suitable for qPCR. Together, the data strongly suggests that *DSV* play a role in the pathogenesis of PD.

*DSV* have an ability to bind to human colonic mucin, and they are found at high levels (approximately 10^4^ to 10^6^ bacteria/g feces) in mucosal samples of the large intestine (Zinkevich and Beech, 2000; Nava et al., 2012; Earley et al., 2015). An important characteristic of *DSV* is its ability to perform dissimilatory sulfate reduction by utilizing sulfate as an electron acceptor for respiration, thereby producing hydrogen sulfide (H_2_S) (Carbonero et al., 2012). H_2_S can also be produced from cysteine degradation catalyzed by L-cysteine desulfhydrase, present in intestinal pathogens such as *Salmonella* Typhimurium, *Helicobacter pylori*, *Escherichia coli* and in pathogens belonging to genera of *DSV, Clostridium, Enterobacter, Klebsiella* and *Streptococcus*. Additionally, *Bilophila wadsworthia* and *D. desulfuricans* can produce H_2_S through a third pathway as a byproduct of taurine catabolism (Carbonero et al., 2012).

H_2_S displays Janus-faced characteristics by carrying physiologic signaling events in neuronal cells and showing neuroprotective properties while also being highly toxic at high concentrations (Panthi et al., 2018; Haouzi et al., 2020). In humans, an acute low-dose H_2_S gas exposition can cause eye irritation and olfactory dysfunction whereas a high-dose exposition can lead to severe central nervous system dysfunction and even death (Rumbeiha et al., 2016; Haouzi et al., 2020). As a diffusible gas that is more soluble than CO_2_ or O_2_, H_2_S can enter the blood circulation from the gut (Tomasova et al., 2016; Haouzi et al., 2020). It is reasonable to assume that H_2_S concentrations are raised in the gastrointestinal wall structures in cases where the gut harbors an increased amount of H_2_S-producing *DSV.* Elevated H_2_S concentrations in these structures may result in constipation due to the compound’s ability to inhibit gastrointestinal motility (Singh and Lin, 2015). In the present study, the constipation prevalence among PD patients was as high as 70%. Notably, constipation is a prevalent ailment in PD and it can precede the motor features of PD and form a risk for PD onset (Abbott et al., 2001; Lin et al., 2014; Stirpe et al., 2016). In one study, constipation was reported to associate with increased quantities of *DSV*, *Cristensenellaceae* and *Firmicute* bacteria in fecal samples of non-PD subjects (Jalanka et al., 2019). However, whether *DSV* species are a cause or a consequence of constipation in PD remains an unanswered question. Possibly, *DSV* species take part in the evolution of PD after their quantity exceeds a certain threshold level.

Hydrogen sulfide has been demonstrated to alter intracellular biochemistry to favor α-Syn aggregation. Hydrogen sulfide can release iron from mammalian ferritin in cells and raise iron levels in the cytosolic labile iron pool (Cassanelli and Moulis, 2001; Hälldin and Land, 2008). The resultant effect on α-Syn-expressing nerve cells is of concern as both ferric and ferrous iron are capable of inducing α-Syn aggregates, the main neuropathologic feature of PD (Joppe et al., 2019). Overexpression of endogenously produced H_2_S can also release mitochondrial cytochrome c into the cytosol, where this cytochrome has been observed to form α-Syn radicals and subsequently induce α-Syn oligomerization, an early stage in α-Syn aggregation (Guo et al., 2015; Kumar et al., 2016; Li et al., 2019). The colonic mucosa is normally protected from H_2_S by the sulfide oxidation pathway, including the enzymes sulfide quinone oxidoreductase, persulfide dioxygenase, rhodanese and sulfide oxidase (Picton et al., 2002; Ramasamy et al., 2006; Libiad et al., 2014). If *DSV*, the dominant SRB in the intestinal mucosa (Zinkevich and Beech, 2000; Nava et al., 2012; Earley et al., 2015), increase in number, H_2_S will likely be produced at higher levels that may exceed the capacity of the detoxifying enzymes. In addition, inflammation decreases the detoxification capacity of the mucosal tissue, resulting in an increased level of H_2_S (Flannigan et al., 2013). The observation that smoking induces a causally protective effect on PD occurrence lends support for the role of H_2_S and its interaction with detoxifying enzymes in PD pathogenesis (Mappin-Kasirer et al., 2020). It is known that cyanide, present in variable amounts in cigarette smoke, reacts with H_2_S under the influence of rhodanese to form thiocyanate, thus resulting in lowered H_2_S levels (Picton et al., 2002).

The enteroendocrine cells of the gut, which display neuron-like properties and are connected to autonomous enteric nerves, express α-Syn (Chandra et al., 2017). Anatomically, enteroendocrine cells extend their apical cytoplasmic processes towards the gut luminal surface. Thus, it is reasonable to argue that this feature will increase the *DSV*-borne H_2_S exposure risk. In addition, overgrowth of *DSV* may induce colonic mucosal barrier dysfunction by influencing the metabolism of butyrate, a short-chain fatty acid (SCFA), which has been reported to be the major energy substance for the colonic epithelium (Chapman, 2001). Overgrowth of H_2_S-producing bacteria such as *DSV* poses an apparent threat to this barrier function, as sulfides impair the oxidation of butyrate (Babidge et al., 1998). In this context, it has been shown that PD patients exhibit increased intestinal permeability correlating with increased intestinal mucosa staining for α-Syn (Forsyth et al., 2011). In addition, lipopolysaccharides produced by *DSV* can apparently increase intestinal permeability and α-Syn expression (Kelly et al., 2014; Fuke et al., 2019; Gorecki et al., 2019). Notably, the mucin layer of the colon consists primarily of glycoproteins, which carry sulfate residues, and degradation products of these sulfomucins serve as a source of sulfate for SRB such as *DSV* (Derrien et al., 2008). Further, *Akkermansia muciniphila* and *Bifidobacterium*, abundant inhabitants of the human gut, can degrade mucin (Derrien et al., 2008; Ruas-Madiedo et al., 2008). Several studies on gut microbiota in PD have shown increases in the relative abundance of these bacteria (Chiang and Lin, 2019; Shen et al., 2021). *Bifidobacteria* are commonly available as commercial products, and their abundance in the gut is reported to correlate to the levodopa dose in PD (Wallen et al., 2020). *A. muciniphila*, in addition to its ability to degrade mucin, seems to promote mucus thickness and stimulate mucus turnover rate, thus apparently freeing considerable amounts of sulfate for SRB (Zhou, 2017). Support for this interaction between *A. muciniphila* and SRB is provided by a study on the metabolome profile of PD patients wherein significant changes in sulfur metabolism, including H_2_S, were verified through computational modeling, and the observed changes were driven by *A. muciniphila* and *B. wadsworthia* (Hertel et al., 2019). As a pathogenetic model, it is justifiable to propose that excessive production of H_2_S by gut *DSV*, cross-fed by *A. muciniphila*, leads to α-Syn oligomerization and aggregation in the adjacent enteroendocrine cells. From there, α-Syn oligomers may make their way to the brain *via* the vagus nerve. This proposed model agrees with the initial proposal by Braak and colleagues that PD is caused by a pathogen capable of passing through the mucosal barrier of the gastrointestinal tract (Braak et al., 2003). Routes other than the vagal route for α-Syn oligomer transport come into consideration as well. Elevated levels of oligomeric α-Syn have been detected in plasma samples of PD patients, and it has been documented that α-Syn can cross the blood brain barrier (BBB) in both the blood-to-brain and brain-to-blood direction (El-Agnaf et al., 2006; Sui et al., 2014). If *DSV-*produced H_2_S plays a central role in the pathogenesis of PD, it is reasonable to presume that, in addition to *DSV*, other H_2_S-producing bacteria, such as *H. pylori* and *Clostridium* species, may also induce PD (Murros, 2021). In fact, people with PD have an increased prevalence of *H. pylori* infections, and eradication of this pathogen has been reported to improve motor functions in PD patients (McGee et al., 2018). Recently, a population-based cohort showed that *Clostridium difficile* infections temporarily elevate the risk of PD (Kang et al., 2020). Although increased production of H_2_S may play a pivotal role in PD pathogenesis, inflammation caused by *DSV* and other infective agents like curli-producing *E. coli* and *Proteus mirabilis* evidently play a role as well (Chen et al., 2016; Choi et al., 2018). Experimentally, an exposure to bacteria that produce the curli protein results in α-Syn depositions in both the gut and the brain (Chen et al., 2016). Furthermore, it has been shown that LPS can accelerate the synthesis of curli fibrils (Swasthi and Mukhopadhyay, 2017). After a primary inflammatory event, a sustained low-level inflammation may develop, resulting in increased intestinal permeability, leakage of inflammatory agents, and ultimately a chronic systemic immune response that may weaken the BBB (Houser and Tansey, 2017).

The potential capability of *DSV* to produce magnetite (Fe_3_O_4_) deserves special attention, as uncoated magnetite nanoparticles can accelerate α-Syn aggregation (Joshi et al., 2015). Most of the *DSV* contain a [FeFe]-hydrogenase metalloenzyme system, which catalyzes both the oxidation and reduction of molecular hydrogen and protons, respectively (Pereira et al., 2011). Based on studies on *D. vulgaris*, it has been suggested that the reduction of soluble ferric iron to ferrous iron is a periplasmic process that requires the presence of a [FeFe]-hydrogenase (Park et al., 2008). An interaction between ferrous iron and amorphous ferric hydroxide can result in magnetite formation, and it has been shown that magnetite can be formed from amorphous ferric hydroxide in the presence of iron- and sulfate-reducing bacteria (Chistyakova et al., 2004; Lenders et al., 2016). *D. desulfuricans* has the ability to synthesize magnetite (Lovley et al., 1993), and this *DSV* species was the most frequently found *DSV* species in the patients included in this study. Notably, magnetite production in anaerobic condition by dissimilatory iron-reducing bacteria is coupled with energy-metabolism and the produced magnetite is extracellular (Konhauser, 1997). Magnetite nanoparticles can be absorbed into intestinal cells and blood circulation by endocytosis (Fröhlich and Roblegg, 2012; Bergin and Witzmann, 2013). In a study on skin samples of patients having PD, low-temperature magnetometric measurements revealed apparent superparamagnetic magnetite particles in the dermal layer of several PD patients, and it was proposed that these particles were probably gut-borne and produced by *DSV* (Murros et al., 2019). Support for the ability of magnetite to accumulate in the brain is provided by a study on 822 brain specimens sampled from seven human cadaver brains (Gilder et al., 2018). However, the possible connection between bacterial magnetite nanoparticles and PD pathogenesis is still speculative; magnetometric data from stool samples and biopsy specimens from the colon and brain of PD patients are currently unavailable.

In the present study, specific *DSV* species were identified in most of the fecal samples of PD, with the quantities of *DSV* correlating with the PD severity. In addition, the *DSV-*specific [FeFe]- hydrogenase gene was found in all PD samples indicating an existence of other unidentified *DSV* species. These findings suggest that *DSV* may be an etiological agent promoting microbiome-related PD pathogenesis. We present the following pathogenetic model. First, *DSV* colonize the intestine permanently, increase in numbers and produce hydrogen sulfide in amounts exceeding the H_2_S detoxification capacity of the mucosal sulfide oxidation pathway (especially the rate-limiting sulfide quinone oxidoreductase), while also producing LPS and magnetite (in at least some *DSV* species) near the enteroendocrine cells. These agents subsequently induce α-Syn oligomerization and aggregation in the intestinal enteroendocrine cells. Secondly, toxic α-Syn oligomers spread in a prion-like manner, traveling from enteroendocrine cells to the brain mainly *via* the vagal nerve and possibly *via* the bloodstream, where they ultimately cause damage to the brain dopaminergic system. In addition, magnetite nanoparticles produced by *DSV* may pass into the bloodstream from the intestine, cross the BBB, and accelerate α-Syn aggregation in the brain. This proposed model should be further evaluated in future research. Future studies could, in the prevention and treatment of PD, focus on developing methods to eradicate *DSV* from the human intestine by antibiotics, phage therapy, fecal transplantation, diet changes, or a combination of these interventions. Isolation of *DSV* from the human intestine is critical, as it allows for designing better primers, antibiotic profiling and phagotype screening. Isolation of these bacteria also enables genome sequencing of PD-associated *DSV* and genomic comparison to environmental and healthy-carrier isolates of *DSV*, potentially aiding in the identification of therapeutic targets among the gene products specific to PD-associated *DSV*.

## Additional Information

Correspondence and requests for materials should be addressed to Kari Murros and Per Saris.

## Data Availability Statement

The raw data supporting the conclusions of this article will be made available by the authors, without undue reservation.

## Ethics Statement

The studies involving human participants were reviewed and approved by Ethics Committee of Helsinki and the Uusimaa Health District area of Finland (“Desulfovibrios in Parkinson’s disease”-protocol was accepted on 27th of May 2019 under the registration number/code HUS/975/22019 and “Occurrence of Desulfovibrio bacteria specific Fe-Fe-hydrogenase gene in feces of patients with Parkinson’s disease” was accepted on 5th of Sept 2019 under the code HUS/2248/2019). The patients/participants provided their written informed consent to participate in this study.

## Author Contributions

KM and VH contributed equally to the work of this study. KM conceived the idea of the PD-*DSV* connection and performed the clinical studies. PS and VH conceived the idea of the *DSV*-specific *hydA*-gene relevance to PD. KM, VH, TT, and PS designed the study. VH performed experiments and analyses under supervision of TT and PS. KM and VH wrote the first draft of the manuscript. TT and PS revised the manuscript, followed by the finalizing of the manuscript by all authors. PS acquired study funding. All authors contributed to the article and approved the submitted version.

## Conflict of Interest

The authors are inventors in a patent application submitted by Helsinki Innovation Services. This patent disclosure (Finnish patent application No. 20205685) is owned by the University of Helsinki.
